# Research progress on moyamoya disease combined with thyroid diseases

**DOI:** 10.3389/fendo.2023.1233567

**Published:** 2023-10-11

**Authors:** Guibo Zhang, Erheng Liu, XueYi Tan, Chengyuan Liu, Shuaifeng Yang

**Affiliations:** ^1^ The Affiliated Hospital of Kunming University of Science and Technology, Kunming, China; ^2^ The First People’s Hospital of Yunnan Province, Kunming, China; ^3^ Department of Neurosurgery, Kaiyuan Municipal People’s Hospital, Kaiyuan, China

**Keywords:** moyamoya disease, thyroid, grave disease, thyrotoxicosis, review

## Abstract

Moyamoya disease (MMD), also known as abnormal cerebral vascular network disease, is characterized by progressive occlusion or stenosis of the internal carotid and cerebral arteries, as well as the formation of an abnormal cerebral vascular network. It can occur anywhere in the world but is most common in China, Japan, and the Republic of Korea. In recent years, there have been increasing reports on the coexistence of thyroid diseases and MMD, but the mechanism of their coexistence is still unclear. For this article, we used keywords such as “moyamoya disease”, “thyroid”, “Grave disease”, “thyrotoxicosis”, and “thyroid autoimmune antibodies” to search for 52 articles that met the requirements in medical databases such as PubMed and Web of Science. This article also reviews the research on the role of thyroid hormone, the mechanism of immune antibodies, the possible correlation between thyroid diseases and MMD disease genes, and the treatment methods, and discusses the possible relationship between MMD and thyroid diseases to provide a reference for the pathogenesis and treatment of MMD with thyroid diseases.

## Introduction

**Figure 1:**
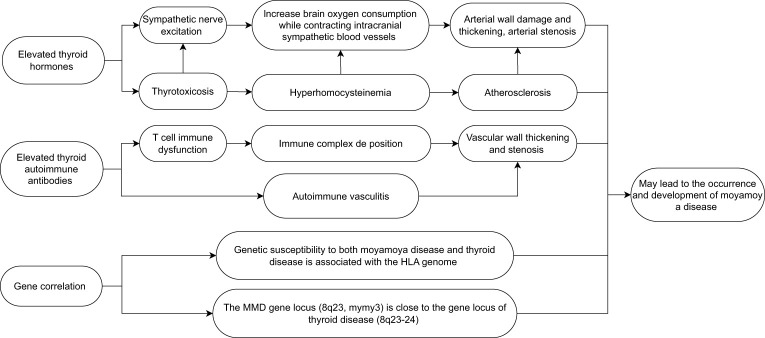
Possible factors leading to the occurrence and development of moyamoya disease caused by thyroid diseases.

Moyamoya disease (MMD) is a rare cerebrovascular disease that was first discovered by Japanese scholars in 1957 (1). It is characterized by progressive occlusion of the superior carotid artery (ICA) and it mainly occurs within the circle of Willis. Occlusion leads to the formation of a compensatory vascular network at the bottom of the brain. The disease is called “moyamoya” (“puff of smoke” in Japanese) disease (2, 3) because it is like a stream of smoke drifting in the air during angiography. The incidence rate of MMD in East Asia is very high, with the greatest number of reported cases coming from China, Japan, and the Republic of Korea (4). Although the cause of MMD is still unclear, in the current research, it is speculated that it may be related to genes, immunity, and external factors. There are reports in the literature that the overall prevalence of autoimmune diseases in MMD patients in western China is as high as 31.0% (5). The characteristic of hyperthyroidism is an increase in the synthesis and secretion of thyroid hormones. Elevated thyroid autoantibodies may also lead to increased hormone levels and secretion. The most common cause of hyperthyroidism is Graves’ disease (6), which is an autoimmune disease. It is characterized by the presence of thyroid hormone-related antibodies (TR-Ab) that stimulate thyroid cells, resulting in the excessive secretion of thyroid hormones. Some patients with Graves’ disease also show an increase in thyroid peroxidase antibodies (TPO-Ab) and anti-thyroid autoantibodies (Tg-Ab) (7, 8). In recent years, many reports have pointed out that there is a correlation between hyperthyroidism and the occurrence and development of MMD (often Graves’ disease is the most common), especially when thyrotoxicosis occurs. The probability of occurrence and progression of MMD is higher than that of hyperthyroidism patients with well-controlled hormone levels (9, 10). However, the mechanism and related treatment of MMD combined with thyroid diseases are not very clear in current research. This article reviews research on the effects of thyroid hormones, immune antibody mechanisms such as TR-Ab, TPO-Ab, and Tg-Ab, the possible association between thyroid diseases and MMD disease genes, and treatment methods, and discusses the possible relationship between MMD and thyroid diseases with a view to providing a reference for the relevant mechanism and treatment of MMD with thyroid diseases (**Figure 1**).

## Literature search

Articles in the PubMed and Web of Science databases were searched to identify articles related to MMD and abnormal thyroid function, with findings being searched up to the year 2022. The titles and abstracts of those articles were reviewed by two reviewers to confirm their quality and eligibility for further examination. The inclusion criteria were as follows: MMD and abnormal thyroid function mentioned together in title or abstract, and original studies or case reports. Other relevant articles and reviews were considered. The final bibliography is based on originality and relevance to the subject. The exclusion criteria were as follows: non-English articles and commentaries or clinical trials.

Based on the inclusion and exclusion criteria, a total of 52 eligible articles were identified and reviewed, and the year of publication ranged from 1997 to 2023. Due to space constraints, we do not look into statistical disaggregation in this report.

## Thyroid hormone

MMD, as a cerebrovascular disease, often leads to serious cerebrovascular accidents due to vascular stenosis or occlusion. Some hormones in the human body can also cause lumen stenosis by contracting the blood vessels. With the continuous progress of research, it has been found that thyroid hormones are closely related to MMD (11). At present, thyroid hormones in laboratory testing usually include serum triiodothyronine (T3), thyroxine (T4), free T4, and free T3 (12). There may be differences across different reports, but the overall results show that the increase in thyroid hormone levels is important in the occurrence or development of MMD. In the study of Asian children with MMD, Hao Li and his team included 114 children with MMD and 114 healthy controls. The incidence rate of increased thyroid function was higher in the MMD group than in the control group; after adjusting for family history of cerebrovascular disease, thyroid function, homocysteine, and other variables, this result is still significant (13). This research result can also be supported by an adult case analysis. From May 2000 to December 2010 Nun Jun analyzed 12 patients from Peking Union Medical College Hospital and four patients from PLA 307 Hospital. In the study, all patients were female, they had an average age of 35.13 years ± 12.34 years (11 years–57 years), and they all had Graves’ disease and MMD. Fifteen patients had cerebrovascular MMD, and the level of thyroid hormone in their bodies increased more significantly than that for patients without the disease (14). Research shows that thyroid hormone can significantly increase the risk of cerebrovascular disease In patients. Yang et al. assessed 16,808 hyperthyroidism patients with thyroid hormone and their probability of cerebrovascular disease. The results show that hyperthyroidism increases the risk of subsequent cerebrovascular disease by 38% (15). Other studies have put forward a similar point of view. After treatment, the blood flow of cerebral vessels in patients with hyperthyroidism will also significantly increase after the thyroid hormone level in their bodies returns to normal (16). Li evaluated the clinical characteristics and treatment effects of 21 patients with both MMD and thyroid dysfunction, and concluded that thyroid dysfunction, especially hormone level fluctuations caused by hyperthyroidism, may be an important risk factor for triggering or exacerbating symptoms in MMD patients (17). This study had a small number of data and further large-scale research may be required. In addition, the occurrence of thyrotoxicosis can further exacerbate the symptoms of patients with MMD. According to reports, a 42-year-old woman with Graves’ disease developed an ischemic cerebral infarction during a thyrotoxicosis attack. The patient’s MRI scan showed typical manifestations of MMD, and high thyroid hormone levels caused by thyrotoxicosis may have been related to the development of MMD in the patient (18). Therefore, when patients with Graves’ disease experience worsening symptoms of cerebral ischemia during follow-up, the possibility of thyrotoxicosis should be considered. As for why the increase of thyroid hormone levels can lead to the occurrence and development of MMD, it is speculated that the following mechanisms may exist: excessive thyroid hormone levels make the sympathetic nervous system more sensitive, increase brain metabolism and oxygen consumption, and at the same time enhance the sympathetic vasoconstriction of the intracranial arteries, leading to the development of artery stenosis and posing a risk to the integrity of the arterial wall (19–21). In addition, thyrotoxicosis may lead to hyperhomocysteinemia, which may also be related to premature atherosclerosis and MMD (22). At the same time, the high level of thyroid hormone caused by the use of exogenous hormones to treat Graves’ disease may also lead to the development of MMD (23). Although more and more studies indicate that an increase in thyroid hormone levels may lead to the development of MMD, the specific mechanism is not very clear, and further research is still needed. At the same time, it is still unclear whether hypothyroidism is related to MMD. More attention should be paid to this issue in the future, so as to better understand the role of thyroid hormone levels in the course of MMD.

## Autoimmune antibody

Multiple reports have indicated that MMD may be related to autoimmunity (TR-Ab, TPO-Ab, Tg-Ab) (24), and the elevation of autoimmune antibodies plays an important role in the development of its course (25). With the development of technology, researchers have found that similar mechanisms may appear in different autoimmune diseases, which can lead to the coexistence both MMD and autoimmune diseases (26). In recent years, more and more literature has shown that autoantibodies produced by thyroid-related diseases also play an important role in the occurrence and development of MMD. Thyroid autoantibodies refer to immunoglobulins produced by autoimmune disorders that target certain components of the thyroid gland. Clinically, there are two main categories: (1) antibodies targeting thyroid stimulating hormone (TSH) receptors on the surface of thyroid cells, namely TR-Ab; and (2) antibodies against thyroid cell contents, including TPO-Ab and Tg-Ab (27, 28). Currently, multiple studies have shown that thyroid autoantibodies may be important triggers for stroke and other cerebrovascular accidents in patients with MMD. After analyzing the prognosis and progression of 37 patients with MMD, Luigi found that the clinical manifestations of MMD patients may be significantly influenced by the concentration of thyroid autoantibodies in the serum, and when the concentration of thyroid autoantibodies in the serum increases, the risk of cerebrovascular accidents in patients also increases (29). At the same time, the study found that the concentration of thyroid autoimmune antibodies in patients with MMD was significantly higher than that in the normal population. In Kim’s study, a total of 63 MMD patients, 71 non-MMD stroke patients, and 200 healthy controls were included. The incidence rate of elevated thyroid autoantibodies in the MMD group was significantly higher than that in other groups. Analysis suggests that thyroid autoimmunity-related or potential immune abnormalities play a role in the development of MMD (30). Leia conducted a similar study, selecting 28 patients with MMD and 28 healthy control group participants. Through a comparative analysis of their thyroid autoantibody levels, it was found that there is a correlation between MMD risk and elevated thyroid autoantibody levels (11). Numerous studies and analyses have shown that thyroid autoantibodies may play a crucial role in the progression of MMD. Although the mechanism of action is still unclear, it can be roughly summarized as the following aspects based on current research: an increase in the level of thyroid autoantibodies in patients may be a part of the reason for the immune imbalance in the MMD system (30). Consistent with this viewpoint, T-cell dysregulation is associated with the cell proliferation and vascular dysregulation observed in MMD. The deposition of autoimmune antibodies or immune complexes, as well as stimulation of blood vessels, leading to the thickening of vascular wall proliferation, may be another cause of MMD (31, 32). At the same time, the cross-reaction between TSH receptor antibodies and cerebral artery antigens can lead to autoimmune-mediated vasculitis. It may also cause the thickening and narrowing of the patient’s blood vessel wall (33). Although there is a significant correlation between the two diseases, there are still some issues that need to be addressed. As MMD is already a rare disease, the number of cases coexisting with thyroid diseases is even rarer, resulting in a generally small sample size for such studies, which may lead to a lack of representativeness in experimental results. At present, literature research on this type of disease is mostly single center reports, and there are differences in sampling methods among different institutions, which may also lead to bias in experimental data. In addition, studies have shown that the increase in autoantibodies caused by Hashimoto’s thyroiditis is also an independent risk factor for cerebrovascular accidents in patients (34). In addition, one of the moyamoya vascular types (MM type) is similar to the clinical manifestations of MMD. However, it is currently unclear whether MM type is an independent entity manifestation related to Hashimoto’s disease or whether it is a true coincidence between MMD and Hashimoto’s disease (35). These issues still need to be investigated by clinical workers.

## Gene correlation

The major histocompatibility complex (MHC) encodes several key immune response genes, which are also known as human leukocyte antigens (HLA) regions in humans. It is located on chromosome 6p21 and the MHC gene, and it can be divided into three regions: MHC Class I, MHC Class II, and MHC Class III. As part of the adaptive immune response, many MHC gene products are involved in the inflammatory response and interact with natural killer cells and cytokines (36). The missense mutation of the gene in RNF213 is independently related to the occurrence of MMD, which has been reported many times (37, 38). In recent studies, it has been found that the genetic susceptibility of MMD is also related to the HLA gene. Wan et al. studied a characteristic cohort of 755 Chinese Han MMD patients and 2,031 healthy control patients. After relevant analysis, it was determined that the common variant rs3129731 within the HLA locus is the main genetic risk factor for obtaining MMD. In their study, a significant association was found between HLA class I and class II genes and MMD risk. These two genes are important components of humoral and cellular immunity, indicating that both types of immunity are involved in MMD. Their team found that the genetic polymorphisms of HLADQA2 and HLA-B may be genetic susceptibility factors for MMD in the Chinese Han population (39). In Japanese patients with MMD, there is a significant correlation between MMD and HLA-DR1 (40); HLA-DRB1 * 1501 and HLA-DQB1 * 0502 are also correlated with MMD (41). Hong concluded after studying the HLA gene in Korean patients with MMD that HLA-DRB1 * 1302 and HLA-DQB1 * 0609 are more likely to show association in familial MMD patients (42). Although there are differences in HLA among patients in Japan, the Republic of Korea, and China, the genetic susceptibility of MMD in the HLA genome has been recognized. In Graves’ disease, the HLA class II DRB1, DQB1, and DQA1 genomes have been confirmed to be related to their occurrence and have demonstrated their induction of DR3 and protection against DR7 (43). At the same time, studies have also shown that the HLA genome is also associated with the occurrence of Hashimoto’s thyroiditis, and HLA plays an important role in the development of thyroid autoimmune diseases (44, 45). At the same time, Tashiro conducted a case-control study on the association between HLA and MMD. Analysis shows that HLA-DRB10410 is a risk allele for MMD, and that this gene is also associated with thyroid diseases in MMD patients. Tashiro’s research proves that there is a possible genetic association be”ween MMD and thyroid diseases (46). In a reported pathological analysis, a mother and daughter were diagnosed with both MMD and Graves’ disease. Tokimura believed that a gene locus (chromosome 8q23, MYMY3) of MMD is very close to autoimmune thyroid diseases (8q23–24), which may lead to the coexistence of the two (47). Although the possible pathogenic genes are also present in the HLA genome and the gene loci are very similar, the gene sequences shown in the article exhibit differences, and data from different regions show different experimental results. Therefore, to clarify the genetic correlation between autoimmune diseases more clearly, more academic research in this area is still needed.

## Treatment

Patients with both MMD and thyroid diseases are currently treated differently from those with simple MMD. Some literature suggests that a patient with both MMD and Graves’ disease was admitted due to acute cerebral infarction. Later, it was found that the patient was in a state of thyrotoxicosis and was discharged after receiving thyroid drug treatment. A few months later, a follow-up examination revealed a significant increase in cerebral blood flow compared with previous cerebral blood flow. It is believed that the treatment of thyroid diseases can effectively delay the development of MMD (48). At the same time, Choi also shared a case in which a patient sought medical attention for thyrotoxicosis accompanied by seizures and ischemic stroke after stopping anti-thyroid treatment for 5 weeks. A brain computed tomography scan diagnosed them as having MMD and the patient’s symptoms were alleviated through surgical bypass surgery. However, after the surgery, the patient developed seizures accompanied by septic cardiogenic shock and mixed atrial fibrillation, ultimately leading to cardiac arrest and death. The author believes that it may be necessary to correct thyroid function to a normal level before undergoing revascularization surgery, which can reduce the risk of perioperative and postoperative complications (49). Endo’s research also supports this conclusion (50). Due to the different clinical treatment methods for elevated thyroid hormones and elevated thyroid autoantibodies, there are also differences in the treatment plans for patients with MMD. In most cases, after controlling hyperthyroidism, the symptoms of cerebral ischemic events caused by hyperthyroidism improved. However, in the cases studied by Ohba, the symptoms of a transient ischemic attack occurred during hypothyroidism rather than hyperthyroidism. Therefore, after excluding variables, they believed that the vascular changes in the cases were caused by immune-mediated mechanisms. It is speculated that for patients with hyperthyroidism accompanied by vascular changes and exacerbation of MMD symptoms, anti-hyperthyroidism treatment may alleviate the condition. However, if the increase in antibodies caused by autoimmune diseases is believed to be mainly related to vascular changes and symptoms, simple anti-thyroid therapy does not seem to have a significant improvement in the symptoms of MMD patients. At this point, surgical treatment may be more beneficial for the patient’s prognosis (31). It may be good for the treatment and prognosis of patients with MMD to use drugs or surgery to reduce the level of thyroid hormones in patients, prevent the occurrence of thyrotoxicosis, and detect and treat autoantibodies, and it needs further research in the future. For patients with severe cerebrovascular symptoms and poor efficacy in simple thyroid treatment, controlling thyroid diseases, using bypass surgery and other methods to restore cerebral blood supply in a timely manner may achieve good results in improving symptoms, quality of life, and long-term disease recovery (51, 52). For MMD patients suspected of concurrent thyroid diseases, timely screening and monitoring of their thyroid function and thyroid autoantibodies, as well as regular follow-up imaging changes, early diagnosis, and selection of appropriate treatment methods based on the situation, may help guide subsequent clinical management and improve patients’ later treatment and recovery.

## Discussion and limitations

With the advancement of medical technology, there are more and more reports of MMD combined with thyroid diseases, but the specific mechanisms and treatments are not very clear (**Table 1**). The reported literature lacks large-scale multicenter prospective research, and the number of studies on molecular mechanisms is also relatively scarce. It is still unclear whether thyroid gland disease may be involved in the occurrence and development of MMD, or whether moyamoya disease patients are more prone to thyroid diseases. In the future, more attention should be paid to this issue, and clinical workers need to increase their research in this area. This may help reveal the pathogenesis of MMD, providing reference for the treatment of thyroid diseases complicated by MMD, guide subsequent clinical management, and improve patients’ treatment and recovery.

**Table 1:** Summary of related studies on moyamoya disease combined with thyroid diseases.

STUDY AUTHOR AND YEAR	CASE NO.	AGE (YEARS)	GENDER	RESEARCH OBJECTIVE	RESEARCH CONCLUSION
Kim Suk Jae, 2010 (30)	63	35–52	M: 37 F: 26	Investigate the association between thyroid autoantibodies and MMD in patients with significantly normal thyroid function.	Thyroid autoimmune or potential immune abnormalities also play a role in the development of MMD.
Li Hao, 2011 (13)	114	6–13	M: 60 F: 54	Are thyroid function and thyroid autoantibodies associated with the risk of MMD in children?	Enhanced thyroid function and elevated thyroid autoantibodies are associated with MMD in children
Lei C, 2014 (11)	28	30–59	M: 10 F: 18	Assess the correlation between elevated thyroid function and elevated thyroid autoantibody levels and the risk of MMD.	The evidence strongly suggests that elevated thyroid autoantibodies and elevated thyroid function are independently associated with MMD.
Chen Jian Bin, 2016 (24)	384	30–50	M: 176 F: 208	Explore whether there is a difference in the prevalence of autoimmune diseases between unilateral and bilateral MMD.	Unilateral MMD may be more associated with autoimmune diseases than bilateral MMD.
Ryu Bike, 2015 (52)	8	29–51	M: 1 F: 7	Discuss the appropriate timing for optimizing thyroid hormone and Graves’ disease MMD surgical treatment.	For MMD with Graves’ disease, optimizing thyroid hormones and performing superficial temporal artery to middle cerebral artery double bypass surgery can successfully prevent cerebral ischemic events.
Lanterna Luigi A, 2018 (29)	37	>18	M: 9 F: 28	Explore the relationship between thyroid autoantibodies and clinical manifestations of MMD.	When the serum concentration of thyroid autoantibodies increases, patients have a higher risk of developing invasive manifestations.
Tashiro Ryosuke, 2019 (46)	136	28–48	M: 11 F: 125	Clarify the association between HLA alleles and MMD.	HLA-DRB1 * 04:10 is a risk allele for MMD, and it is also associated with thyroid diseases in MMD patients.

M represents male and F represents female.

## Author contributions

GZ, EL, XT and CL contributed equally to this work. All authors contributed to the article and approved the submitted version.
